# Endoscopic submucosal dissection of gastric neoplasms with severe fibrosis using a new thin-therapeutic endoscope and a dedicated conical cap

**DOI:** 10.1055/a-2106-0688

**Published:** 2023-07-13

**Authors:** Yoshimasa Miura, Hisashi Fukuda, Takashi Ueno, Yoshikazu Hayashi, Hiroyuki Osawa, Alan Kawarai Lefor, Hironori Yamamoto

**Affiliations:** 1 Department of Medicine, Division of Gastroenterology, Jichi Medical University, Shimotsuke, Tochigi, Japan; 2 Department of Surgery, Jichi Medical University, Shimotsuke, Tochigi, Japan

Recently, endoscopic submucosal dissection (ESD) using the pocket-creation method (PCM) has been developed as a kind of third space endoscopy. PCM enables complete dissection under the lesion before making the circumferential incision 1 . Using this method, a wide submucosal space should be created with a small entrance where a small-diameter endoscope is more desirable.

A few endoscopists reported ESD using ultrathin endoscopy, but disadvantages for such a procedure have been discussed. Although the accessory channels of existing ultrathin endoscopes (EG-L580NW7, Fujifilm Co., Tokyo, Japan) have a diameter of 2.4 mm, some ESD devices such as the knife and coagulation forceps cannot be inserted through the channel because of their larger diameter 2 .

The EG-840TP (Fujifilm Co.) was newly developed for endoscopic treatment as a relatively thin endoscope that has an outer diameter of only 7.9 mm but an accessory channel diameter as large as 3.2 mm ( Fig. 1 ). It also has a down angle function up to 160 degrees. A calibrated, small-caliber-tip, transparent hood (CAST hood; TOP, Tokyo, Japan) was designed to perform balloon dilation of small intestinal strictures 3 . It is transparent with a 4-mm tip diameter that can be used in ESD. PCM combined with this hood may be more effective in cases with gastric neoplasms accompanied by fibrosis 4 .

**Fig. 1:**
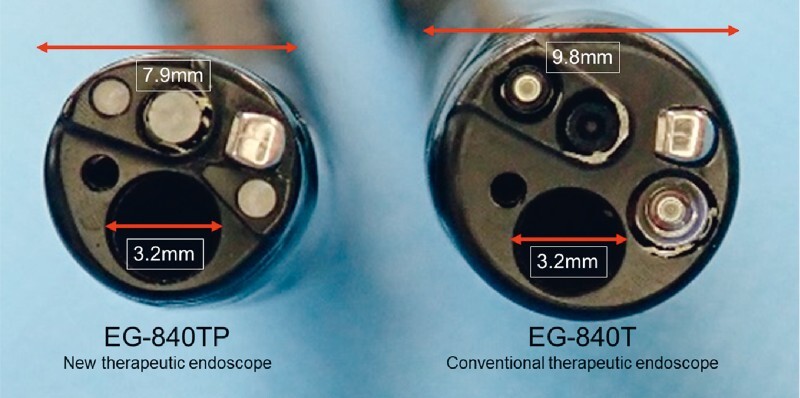
The EG-840TP manufactured by Fujifilm Corporation was recently developed for endoscopic treatment as a thin endoscope that has an outer diameter of only 7.9 mm compared to 9.8 mm of a conventional therapeutic endoscope (EG-840 T); it maintains an accessory channel diameter of 3.2 mm.

We present a patient with gastric cancer with fold convergence that recurred after ESD, located in the posterior wall of the middle gastric body. ESD was performed using the PCM with the EG-840TP and a dedicated conical cap (prototype CAST hood), resulting in successful complete resection. Such a therapeutic approach is expected not only to allow precise dissection of severely fibrotic tissue but also to improve endoscopic maneuverability even in a narrow submucosal pocket with severe fibrosis ( Video 1 ). This endoscope may be used for third space endoscopy especially in a narrow space.

**Video 1** The procedure for the pocket-creation method with an EG-840TP and a dedicated conical cap for early gastric cancer.


Endoscopy_UCTN_Code_TTT_1AO_2AG

## Notes

Endoscopy E-Videos

https://eref.thieme.de/e-videos
*E-Videos* is an open access online section of the journal *Endoscopy* , reporting on interesting cases and new techniques in gastroenterological endoscopy. All papers include a high-quality video and are published with a Creative Commons CC-BY license. Endoscopy E-Videos qualify for HINARI discounts and waivers and eligibility is automatically checked during the submission process. We grant 100% waivers to articles whose corresponding authors are based in Group A countries and 50% waivers to those who are based in Group B countries as classified by Research4Life (see: https://www.research4life.org/access/eligibility/ ). This section has its own submission website at https://mc.manuscriptcentral.com/e-videos
